# An Adaptive Temporal Convolutional Network Autoencoder for Malicious Data Detection in Mobile Crowd Sensing

**DOI:** 10.3390/s24072353

**Published:** 2024-04-07

**Authors:** Nsikak Owoh, Jackie Riley, Moses Ashawa, Salaheddin Hosseinzadeh, Anand Philip, Jude Osamor

**Affiliations:** 1 Department of Cyber Security and Networks, School of Computing, Engineering and Built Environment, Glasgow Caledonian University, Cowcaddens Road, Glasgow G4 0BA, UK; 2 School of Computer Science and Engineering, University of Westminster, 309 Regent Street, London W1B 2HW, UK

**Keywords:** mobile crowd sensing, autoencoders, internet of things, deep learning, temporal convolutional networks, malicious data detection

## Abstract

Mobile crowdsensing (MCS) systems rely on the collective contribution of sensor data from numerous mobile devices carried by participants. However, the open and participatory nature of MCS renders these systems vulnerable to adversarial attacks or data poisoning attempts where threat actors can inject malicious data into the system. There is a need for a detection system that mitigates malicious sensor data to maintain the integrity and reliability of the collected information. This paper addresses this issue by proposing an adaptive and robust model for detecting malicious data in MCS scenarios involving sensor data from mobile devices. The proposed model incorporates an adaptive learning mechanism that enables the TCN-based model to continually evolve and adapt to new patterns, enhancing its capability to detect novel malicious data as threats evolve. We also present a comprehensive evaluation of the proposed model’s performance using the SherLock datasets, demonstrating its effectiveness in accurately detecting malicious sensor data and mitigating potential threats to the integrity of MCS systems. Comparative analysis with existing models highlights the performance of the proposed TCN-based model in terms of detection accuracy, with an accuracy score of 98%. Through these contributions, the paper aims to advance the state of the art in ensuring the trustworthiness and security of MCS systems, paving the way for the development of more reliable and robust crowdsensing applications.

## 1. Introduction

Mobile crowd sensing (MCS) is a paradigm that involves leveraging the ubiquitous presence of mobile devices, such as smartphones, tablets, and wearables, to collect data from their built-in sensors and user inputs [1]. It entails the engagement of mobile users who serve as contributors, operators, or consumers, supplying sensing data to an MCS platform [2]. By outsourcing sensing activities to individual users, MCS lowers the deployment and maintenance costs compared with conventional sensor networks [3]. The main idea behind MCS is to encourage people to perform activities and submit data using smart devices (such as smartphones and mobile wearables) equipped with sensors that collect tons of valuable data.

Mobile crowd sensing has emerged as a prominent paradigm for collecting data from many mobile devices, enabling a wide range of applications, such as environmental monitoring [4], urban planning [5], and healthcare [6]. MCS empowers citizens to participate in data collection efforts by rewarding them for their contributions, leading to smart and sustainable spaces [7].

In the field of mobile crowd sensing (MCS), many issues need careful consideration and creative solutions must be developed. Primarily, privacy and security concerns in MCS systems pose significant obstacles, requiring strong safeguards to protect user data from unwanted access [8]. Furthermore, the task of guaranteeing the dependability and accuracy of crowdsensed data presents significant challenges due to the inherent anonymity and lack of consistency across data sources. The continuous energy consumption linked to MCS operations presents an additional substantial challenge, endangering the battery longevity of mobile devices at a concerning rate. Equally significant is the need to provide incentives for active engagement in MCS initiatives.

Formulating efficient pricing techniques, correcting price disparities, and creating appealing incentive systems pose complex issues. The recruitment and retention of participants in multimedia content creation (MCS) initiatives present complex challenges since dropout rates increase when people perceive inadequate incentives or are excluded from the selection procedures [9]. The management of the many duties performed by users in MCS campaigns introduces an additional level of complexity, especially when considering different priorities and processing capabilities. Moreover, the increasing popularity of mobile crowd sensing highlights the urgent issue of malicious data injection. Engaging in such malicious behaviors has the potential to significantly compromise the precision and reliability of gathered data, thereby hindering the capacity to conduct precise analysis and make informed decisions [10]. When faced with this significant obstacle, the use of autoencoders presents itself as a potentially fruitful approach. An autoencoder neural network is an unsupervised learning framework with an input layer, one or more hidden layers, and an output layer. Architecturally, autoencoders efficiently learn compressed representation with small hidden layers [11]. Autoencoders can enhance the integrity of MCS systems in the face of malicious intrusions by effectively acquiring compressed representations.

Recently, temporal convolutional network autoencoders (TCN-AE) have been proposed for anomaly detection for the Internet of Things [12,13]. TCN-AE has a deep neural network architecture composed of an encoder and a decoder. The encoder comprises convolutional layers. The reverse of the encoder is the decoder, which consists of deconvolutional layers [14].

Despite the promising capabilities of autoencoders in anomaly detection, their use in detecting malicious data in MCS remains unexplored. This paper proposes an adaptive temporal convolutional network autoencoder-based model (ATCN-AE) for malicious data detection for mobile crowdsensing applications. The model combines the strengths of temporal convolutional networks (TCN) and autoencoders to effectively capture and detect malicious data patterns in the temporal sequences collected from mobile devices.

The temporal nature of the data collected in mobile crowdsensing applications presents unique challenges for detecting malicious activities. Temporal convolutional networks (TCNs) have shown great promise in capturing temporal dependencies and patterns in sequential data. By leveraging dilated convolutions, TCNs can capture long-range dependencies, enabling them to extract relevant features from the temporal sequences [15]. Complementing the TCN component, an autoencoder is employed to further enhance the TCN-AE performance in detecting malicious data. The proposed model incorporates an adaptive learning mechanism to adapt to the dynamic nature of the mobile crowdsensing environment. Adaptive learning enables the model to continuously update its parameters and adapt to new patterns or emerging malicious activities, hence ensuring that the model remains effective over time, even as the characteristics of malicious data evolve. The main contributions of this paper are as follows:

1. To introduce a novel ATCN-AE model specifically designed for detecting malicious data in MCS. The ATCN-AE leverages the power of adaptive temporal convolutional networks and autoencoders to effectively capture and analyze temporal patterns in MCS data, enabling the accurate identification of malicious activities.
2. The proposed ATCN-AE model demonstrates exceptional learning capabilities, enabling it to adapt to the dynamic nature of MCS data. By incorporating adaptive temporal convolutions, the model can effectively capture temporal dependencies and anomalies in the data, enhancing its ability to identify and classify malicious instances accurately.
3. The performance of the proposed ATCN-AE model is evaluated using a publicly available dataset relevant to MCS. The experiments conducted on the dataset reveal that the model effectively detects malicious activities in MCS scenarios.

The remainder of the paper is organized as follows. Section 2 reviews autoencoder-based anomaly detection models in IoT and MCS systems. The section also presents some works that employ the SherLock dataset for machine learning model training and evaluation. Section 3 of the paper introduces the proposed model architecture and methodology. It details the adaptive temporal convolutional network autoencoder model, including the input, detection, and output components. The training and evaluation process is also outlined. Section 4 presents the experimental results and discussion. It evaluates the performance of the proposed ATCN-AE model on the dataset using various metrics. The results are analyzed and compared with existing baseline models. The section examines how well the model can detect malicious data in mobile crowdsensing applications. Section 5 draws conclusions and outlines future work.

## 2. Related Works

In this section, we examine the related literature on proposed systems and techniques for anomaly detection in IoT and MCS. This section specifically reviews the use of deep learning models in detecting attacks and other malicious activities in sensing data.

### 2.1. Anomaly Detection Using Deep Learning

In [16], Khanam et al. presented a classwise focal loss variational autoencoder (CFLVAE), a deep generative-based model, to solve unbalanced network traffic problems in intrusion detection systems for the Internet of Things (IoT). A well-balanced intrusion dataset is used to create fresh samples for minority attack types and train a deep neural network (DNN) classifier, improving intrusion detection accuracy. The CFLVAE-DNN has a 3.77% false positive rate and 88.08% intrusion detection accuracy. The suggested model detects low-frequency attacks for U2R (79.25%) and R2L (67.5%). The results show that the model increases learning-based classifier intrusion detection accuracy and handles unbalanced network traffic in IoT intrusion detection systems. However, the approach may not be adequate for real-time intrusion detection systems due to data creation time.

Similarly, Lahasan et al. [17] put forward an optimized deep autoencoder model for anomaly detection in IoT devices with limited hardware support. The authors propose an optimized deep autoencoder model for intrusion detection. The model optimization is performed in two layers. The first layer selects the most relevant input features, training instances, and several hidden neurons simultaneously. The second layer develops the optimized model by combining the accuracy of a K-nearest neighbors (KNN) classifier with the complexity of the autoencoder architecture. The model is evaluated on the N-BaIoT intrusion detection dataset and compared with other optimization algorithms, including the arithmetic optimization algorithm (AOA), particle swarm optimization (PSO), and reinforcement learning-based memetic particle swarm optimization (RLMPSO). The optimized deep autoencoder achieves 99% anomaly detection accuracy with a lightweight model architecture. On average, only 30 input features and 2 hidden neurons are required. The model presented by the authors demonstrates superior performance compared with the benchmark algorithms. The two-layer optimization strategy enables the development of an accurate yet low-complexity intrusion detection model. Nonetheless, the authors do not provide any information about the computational resources required to train and test the model.

Salahuddin et al. [18] introduced a new time-based anomaly detection system called *Chronos* to detect distributed denial of service (DDoS) attacks. *Chronos* employs an autoencoder model trained on time-based features extracted from network packets. The features are aggregated over varying time windows to characterize different DDoS attacks. The CICDDoS2019 dataset containing DDoS attacks via TCP and UDP application layer protocols is utilized. The impact of different time windows in extracting distinguishing features for each DDoS attack type is evaluated. The authors examined how effectively the time-based autoencoder approach can leverage these temporal features to detect and classify DDoS attacks. However, the authors do not compare *Chronos* with other time-based anomaly detection systems to benchmark performance.

Thill et al. [12] proposed an unsupervised anomaly detection algorithm called TCN-AE that utilizes dilated convolutions to learn temporal patterns in time series data. The TCN-AE model comprises a temporal convolutional network (TCN) combined with an autoencoder architecture. The authors evaluated the algorithm on electrocardiogram (ECG) recordings from patients with cardiac arrhythmia. However, the TCN-AE model is only tested on this single dataset of ECG signals. The limited evaluation dataset makes it difficult to assess how well the approach generalizes to other types of time series data, such as MCS.

In [14], Aloul et al. proposed a novel intrusion detection model combining adversarial autoencoders (AAE) and K-nearest neighbors (KNN) classification. The model is designed for deployment on resource-constrained IoT edge devices like small routers. The authors employed the synthetic minority oversampling technique (SMOTE) to balance the class distribution of the NSL-KDD dataset used for evaluation. The AAE performs representation learning on the preprocessed data. The KNN algorithm then leverages these learned features to classify network traffic as benign or anomalous. The model achieves 99.91% accuracy on the NSL-KDD dataset. Nevertheless, the complexity of real-world IoT networks with numerous connected devices may impact the performance of the model. The scalability of the approach concerning increased network traffic and evolving attack types also requires further analysis.

Also, Yang et al. [19] presented an autoencoder-based framework for DDoS attack detection. The methodology consists of two key components—the feature extraction component (FEC) and the online detection component (ODC). The FEC uses the Data Plane Development Kit (DPDK) to extract relevant traffic features from raw packet data. The ODC then leverages the autoencoder model to analyze each sample and identify anomalies based on their reconstruction error. The framework only requires benign traffic to train the detection model, which can automatically update itself. Experiments on synthetic and public datasets demonstrate an 82.0% detection rate and a 0% false positive rate, outperforming classical detection approaches. The model can also detect zero-day and unknown DDoS attacks. Three datasets are utilized for evaluation: a synthetic dataset with over 38,000 attack types, the UNB 2017 dataset, and the MAWI dataset. However, the model may fail to detect DDoS attacks and payloads using encrypted traffic.

Likewise, Yang et al. [19] proposed an unsupervised AE framework that detects DDoS and zero-day attacks in IoT networks. The framework comprises a feature extraction component (FEC) and an online detection component (ODC). These components perform feature extraction and anomaly detection, respectively. Lee et al. [20] employed edge computing with a deep autoencoder model to detect impersonation attacks. The model uses feature abstraction and iterative gradient-based optimization to update its parameters. The authors evaluated the model with the AWID dataset. AWID contains simulated real-world wireless network data with novel attack types, making it well-suited for evaluating IoT intrusion detection systems. Experiments revealed that the model achieves 99.9% accuracy and 99.9% detection rate. The unique wireless simulation environment and incorporation of new impersonation attacks in AWID are critical for developing effective detection models for modern IoT networks. However, the model requires a large amount of training data to achieve high accuracy, which may not be feasible in some scenarios.

Using optimum thresholding, Dhole et al. [13] classified video events as anomalies or benign data. The authors employed a convolutional LSTM autoencoder for the classification. The model has two components: a spatial autoencoder and a temporal encoder-decoder. The spatial autoencoder learns the structural patterns within each video frame, encoding the spatial relationships. Reconstruction errors in the proposed model are reduced via backpropagation. Events are classified as benign when the reconstruction error is less than the optimum threshold and anomaly when it is otherwise.

On the other hand, a framework for anomaly detection in industrial computer systems (ICS) called KingFisher is presented by Bernieri et al. [21]. The framework uses a probabilistic VAE (variational autoencoder) to track network traffic and real-time conditions of physical devices at several network points. KingFisher analyzes single modules and their correlated data to detect anomalies but requires no prior knowledge of the physical model. In the training phase, the framework automatically learns the benign behavior of the physical data.

In detecting anomalous network communications in IoT networks, Shahid et al. [15] presented a sparse autoencoder for detecting anomalous network traffic in IoT systems. The model aims to differentiate between legitimate and malicious communications. Bidirectional TCP flows are extracted to characterize benign network behavior. Statistics on packet sizes and inter-arrival times for the first N packets are calculated as input features. The sparse autoencoders are trained on data from an experimental smart home network to learn patterns of benign communications. Malicious flows for testing are obtained from the IoTPOT honeypot infected with IoT malware. The model achieves attack detection rates of 86.9% to 91.2% and low false positive rates from 0.1% to 0.5% on the test data. Nonetheless, the approach may have difficulty detecting attacks using more sophisticated evasion techniques resembling benign traffic patterns.

Similarly, Meidan et al. [22] presented an anomaly detection method called N-BaIoT for detecting compromised IoT devices using deep autoencoders. The four-stage methodology involves data collection, feature extraction, anomaly detector training, and continuous monitoring. The autoencoders are trained on statistical features extracted from benign network traffic. The model is applied to new data from an IoT device to identify anomalies. The model captures raw traffic data by mirroring switch ports in an organization’s network. The authors evaluated N-BaIoT by infecting nine commercial IoT devices in a lab environment with Mirai and BASHLITE botnets. The model accurately detected the attacks launched by the infected bots, achieving a 99.99% true positive rate and 0.0001% false positive rate. In contrast, the approach may fail against advanced evasion attacks designed to bypass anomaly detectors. It also requires significant computational resources unsuitable for resource-constrained IoT devices.

Meanwhile, Wang et al. [23] developed a new anomaly detection approach called S2-VAE for video data. It comprises two variational autoencoder models: stacked fully connected VAE (SF-VAE) and skip convolutional VAE (SC-VAE). The SF-VAE is a shallow network that models the true data distribution as a Gaussian mixture. The SC-VAE is a deeper generative model leveraging CNN, VAE, and skip connections to learn video representations. The S2-VAE is evaluated on four public datasets: UCSD, Avenue, UMN, and PETS. It is compared with Conv-AE and other state-of-the-art methods like Sparse, MDT, SF, MPPCA, and MPPCA + SF. Experiments show improved performance by S2-VAE in detecting local abnormalities in individual frames and global anomalies across longer video sequences. Combining a simple distribution modeling network and a deeper convolutional-VAE architecture enables robust video anomaly detection.

### 2.2. Anomaly Detection in Mobile Crowd Sensing

In [24], Mohammed et al. implemented a deep learning system that ensures target localization in an error-prone environment. The three-phase approach comprises mean reconstruction (MR), anomaly detection and correction (ADC), and convolutional denoising autoencoder (CDAE). The model is validated in a radioactive setting using anomalous sensor readings from randomly distributed nodes of interest. It achieves 5 times as much accuracy, 50 times as much speed, and 3 times as much energy efficiency compared with existing models. Notwithstanding, multi-sensor fusion is not explored for a full localization framework. The approach focuses only on single-sensor data. Extending the model to integrate multimodal measurements could further enhance localization accuracy and redundancy against individual sensor failures.

Hameed et al. [4] formulated an IOTA-based methodology using machine learning algorithms to detect and prevent fake sensing activities in mobile crowd sensing. The methodology involves two platforms: IOTA and Logit-boosted models. The Logit-boosted models were applied to the IOTA bottleneck dataset and the new IoTA-Botnet 2020 dataset to demonstrate the model’s performance in detecting fake sensing activities. Multiple logit-boosted algorithms were used, including Logi-XGB, Logi-GBC, Logi-ABC, Logi-CBC, Logi-LGBM, and Logi-HGBC. The authors used the IOTA bottleneck dataset to evaluate the proposed model. Evaluations reveal that the Logi-CBC algorithm outperformed the other algorithms regarding accuracy on the given dataset, achieving a detection accuracy of 99.8%. The results suggest that the proposed methodology can be used for quality estimation and incentive allocation in mobile crowdsensing systems. However, the potential limitations of implementing the model in real-world mobile crowdsensing systems are not discussed.

On the other hand, Alharam et al. [25] evaluated classical machine learning models for classifying sensor data as true readings, faulty sensor errors, or malicious attacks. The algorithms examined included a decision tree (DT), support vector machine (SVM), and random forest (RF). The authors emphasized the importance of data preprocessing techniques, like data cleaning, feature selection, and normalization, to improve model generalization. The preprocessed data are used to train the classifiers. Experiments are conducted on a solar radiation dataset from a 200 m × 200 m area with 121 uniformly distributed sensors. Among the evaluated models, random forest achieves the highest accuracy of 97.9% on this dataset. However, deep learning techniques were not explored by the authors. Testing more advanced deep neural networks could potentially improve the reported results.

A hybrid approach combining deep learning and classical machine learning is introduced for detecting and filtering out false data points in mobile crowdsensing (MCS) systems by Afzal-Houshmand et al. [26]. The proposed approach is called FSD (forecasting-based sensor data filtering). The authors use real and simulated datasets to evaluate the performance of the proposed solution under various attack scenarios. The real dataset is collected from a mobile sensing platform called *SensingBus*, which is a mobile sensing platform that collects data from various sensors on mobile devices. The simulated dataset is generated using the same statistical properties as the real dataset. The evaluation metric used is F-Measure, which considers true/false positive rates and recall and precision metrics. Analysis indicates that the solution outperforms existing resilient aggregation and outlier detection schemes.

Similarly, Munoz-Organero et al. [27] presented a model for automatically detecting street elements such as traffic lights, street crossings, and roundabouts using GPS data from a mobile device while driving. The methodology involves preprocessing GPS data to derive speed and acceleration–time series, an outlier detection algorithm to identify normal driving locations, and deep learning-based analysis of speed and acceleration patterns at each outlier to extract relevant features. Features are classified into traffic lights, street crossings, and urban roundabouts. The model achieved a combined recall of 89% and a combined precision of 88% for classifying elements belonging to any of the three target classes in the first dataset. For the second dataset, the model achieved a combined recall and precision of 82%. The proposed model uses an automatic feature extraction mechanism based on a DBN (deep belief network) and a final classifier based on KNN and SVM.

Venkatesh et al. [28] presented a machine learning framework aimed at detecting malware and classifying data theft in smartphones using Android usage data collected via the SherLock framework. The architecture employs supervised tree-based models like Extra Trees, random forests, decision trees, and XGBoost for malware detection, along with an isolation forest for anomaly detection. Performance evaluation involves metrics like accuracy for malware detection and F1 score for data theft classification. Data preprocessing techniques are utilized to condense the feature set, ensuring model robustness across multiple users and diminishing training data. The architecture can classify the type of data being stolen with 83% certainty. However, the study overlooks a detailed analysis of the preprocessing methods used. Also, concerns arise regarding the effectiveness of unsupervised methods in detecting malicious activity due to observed overlaps in density distributions of crucial features.

Similarly, Memon et al. [29] explored Android malware detection using seven distinct machine learning classifiers, including gradient-boosted trees, modified SVM kernels, logistic regression, Bayes Net, and naive Bayes. Their study employed the SherLock dataset, renowned for its size and comprehensiveness. Experiments were conducted on a 17-node Apache Spark cluster, where gradient-boosted trees exhibited the lowest false positive rate (9.2%) and superior precision across benign and malicious labels. Notably, tree-based methods outperformed others in F1 score with an 81.0% accuracy, while SVM showed subpar performance. However, the accuracy recorded by the authors is low, which is mostly because of the shallow machine learning algorithms employed.

In [30], Zheng et al. identified optimal feature sets and methods for mobile app malware detection and prediction of app type and running state. Random forest emerges as the top-performing method for both app classification and malware detection with a 91% accuracy, closely followed by ANN and LSTM. LSTM excels with block statistics as features but lags in malware detection, suggesting limited sequential patterns in running states. Temporal features enhance classification performance, with block-based usage statistics being the most effective. Multi-label classification methods, accounting for label correlations, yield marginal improvements. The study utilizes the SherLock dataset, capturing ongoing attacks within low-privileged monitorable features. However, the focus on usage behaviors and temporal patterns for prediction overlooks other potential contributors to malware detection. While evaluating various classification techniques, the paper lacks comparisons with existing benchmarks, hindering the assessment of its competitiveness.

Existing studies have proposed various models for detecting malicious activities in MCS, primarily employing shallow machine learning algorithms [24,25,26,27,28,29]. The authors of [30] utilized a long short-term memory (LSTM) for malware detection in Android applications. However, these prior works have not addressed the challenge of identifying potential malicious data when sensors such as accelerometers, gyroscopes, and magnetic fields are leveraged in mobile crowdsensing (MCS) activities.

Furthermore, the literature lacks an adaptive model capable of recognizing evolving patterns and detecting novel malicious data in the context of MCS. This gap is significant as MCS systems rely on the collective contribution of data from numerous mobile devices, making them susceptible to adversarial attacks or data poisoning attempts that could compromise the integrity and reliability of the collected information.

Addressing this gap, our paper proposes an adaptive model based on temporal convolutional neural networks (TCNs) for detecting malicious data in MCS scenarios involving sensor data from mobile devices. The proposed TCN-based model aims to address the limitations of existing approaches by providing an adaptive and robust framework that can identify and mitigate potential threats in real time, ensuring the trustworthiness and security of MCS systems. Table 1 summarizes the anomaly detection system reviewed in this section.

## 3. Methods

**Figure 1:**
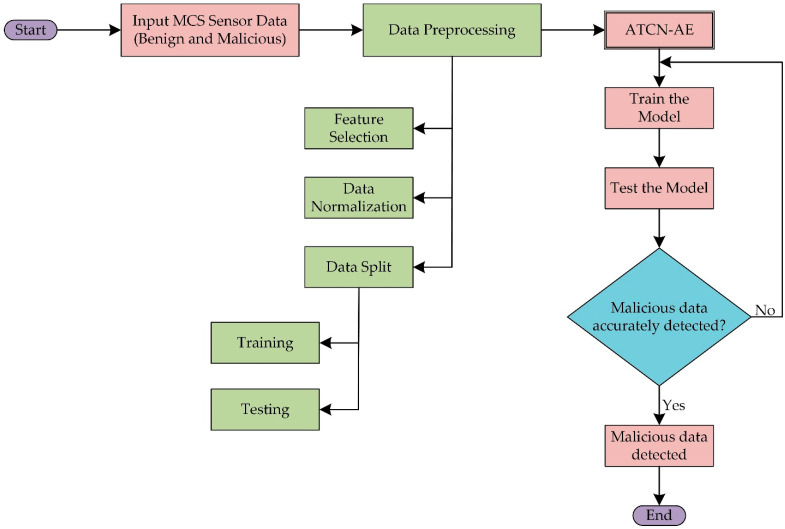
The flowchart of the proposed adaptive temporal convolutional network autoencoder (ATCN-AE).

In this paper, we perform malicious data detection on mobile crowdsensing activities while adaptively learning the sensor data from smartphones. The following steps are involved in the proposed approach, as shown in Figure 1.

### 3.1. Dataset

The SherLock dataset [31] was utilized to validate our proposed model, offering a vast repository of over 10 billion records, totaling six terabytes of data, accumulated from 50 volunteers across several years. This dataset, sourced from Samsung Galaxy S5 smartphones, encompasses time series data from various sensors alongside contextual details like device location, motion, and battery usage. Notably, data collection encompassed benign and malicious operations of the “Moriarty” application, with the latter involving time-stamped attack activities, thus furnishing labeled malicious samples alongside benign data. This controlled environment allows for the systematic recording and analysis of malicious behaviors, enhancing the dataset’s utility for understanding such activities in Android smartphones.

This enriched dataset facilitated the development of models adept at identifying anomalous behaviors rooted in sensor patterns and contextual cues. Conversely, benign operations contributed to the dataset with benign data. The dataset shows diversity in both sensing device models and the types of data recorded. It includes resource utilization snapshots per running app, captured at high resolutions, as well as other smartphone usage aspects such as call logs, SMS, Wi-Fi, and location data.

Moreover, the dataset’s utility extends to various applications, including basic malware analysis and continuous user authentication method evaluation. With readings from seven PUSH sensors and five PULL sensors, our focus was on utilizing the T2 PUSH sensor data for model evaluation. As shown in Table 2, this subset, containing an accelerometer, gyroscope, magnetic field, rotation vector, and barometer sensors, along with metadata like uuid, userid, version, and timestamp, comprised 238 sensor features across thousands of records. For our experiments, the initial 1325 records were utilized for model training and evaluation.

Combining information from both Moriarty and T2 datasets, labeled data points were generated, specifically those including T2 instances from Moriarty application sessions featuring both benign and malicious data. This combined dataset was then integrated into the data frame using user, start and end timestamps, and session identifiers, providing comprehensive insights into each T2 record explaining the specific actions or sessions conducted by Moriarty during data observations.

### 3.2. Preprocessing

Prior to training our proposed model, we conducted data preprocessing to eliminate noisy or irrelevant data points, a common practice in deep learning workflows to enhance model performance. The autoencoder framework, comprising encoder and decoder components, was employed for dimensionality reduction and feature selection. Specifically, the encoder compressed the high-dimensional input data into a lower-dimensional latent representation. The size of the latent space, often referred to as the bottleneck layer in autoencoders, dictates the extent of dimensionality reduction achieved. Our proposed autoencoder retains only the most pertinent features or combinations of features. By reducing the dimensionality of the latent space, the autoencoder effectively sifts through the input data, discarding noisy or irrelevant features while preserving those that contribute most significantly to the underlying data structure. Following this process, the selected features consist of data from the accelerometer, gyroscope, and magnetic field sensors.

Continuing with our preprocessing approach, we took steps to ensure that the selected features were on a consistent scale. This process was conducted to improve the convergence and effectiveness of the model during training. To achieve this, we employed Min-Max normalization, which scales the data to a predefined range of 0 to 1. The Min-Max equation is depicted in Equation (1).

(1) $$X\_normalised=\frac{X-X\_min}{X\_\max-X\_min}$$

where X is the original feature value, X_min is the minimum value of the feature. The feature values are transformed to a range of 0 and 1 by subtracting the minimum value and dividing by the range.

We ensured that the optimal range of data values was picked to represent benign data by randomly selecting the training and testing data. After that, the first 40,000 data entries on the shuffled benign data were concatenated with the malicious to create the training and testing dataset. The timestamp of each data is used to sort the dataset. We employed the percentage split method to evaluate the performance of the proposed model. The dataset was partitioned into two subsets: 80% of the data was allocated to the training set, while the remaining 20% constituted the test set. The model was trained on the training dataset, and its performance was subsequently evaluated on the unseen sensor data from the test set. This approach enabled us to obtain an estimate of the model’s generalization capability on previously unseen data samples. The percentage split method was selected for its computational efficiency as it required training the proposed model only once, thereby reducing the overall computational overhead.

While the percentage split method is subject to potential bias introduced by a single train-test split, it was deemed appropriate for the proposed model due to the substantial size of the T2 file containing the data from the selected features (accelerometer, gyroscope, and magnetic field sensors) in the SherLock dataset.

### 3.3. The Proposed Model

In this section, we delve into the details of our model, offering insights into the algorithm of the proposed ATCN-AE model, its architecture, and the constituent components that comprise it.

#### 3.3.1. Preliminaries

The notation and the associated description used in the ATCN-AE are given in Table 3. It provides a comprehensive overview of the symbols and their meanings. The autoencoder is trained to reconstruct the input pattern at the output stage of the network. The ATCN-AE accepts input $X\in Z^{d}$ and initially maps it to the hidden layer $h=f_{\Theta }=\sigma (WX+b)$ with the parameters $\Theta =\left\{W,b\right\}.$ A reverse mapping of $f:y=f_{\Theta }(h)=\sigma (\bar{W}h+b)$ with $\Theta^{\prime }=\{\bar{W},b^{\prime }\}$ is employed for input reconstruction. The parameters define the encoder W, learned from the hidden layer to the output layer. The relationship between the decoder and the parameters in the encoder can be represented by $\bar{W}=W^{T}$ [32]. The ATCN-AE employs the backpropagation algorithm to minimize the reconstruction error e between each input $x_{t}$ and its associated output $y_{i}$ by altering the parameters of the encoder W and the decoder, $\bar{W,}$ as presented in Equation (2).

(2) $$e(X,y)=\frac{1}{2N}\sum_{i}^{N}t{\left\|X_{i}-y_{i}\right\|}_{2}^{2}$$

Let $S=(s_{1},s_{2},...,s_{n}{)}^{T}=(s^{1},s^{2},...,s^{t})\in R^{n\times t},$ represent a multivariate time series dataset and n features where t denotes the timestamp of each value. We use $st=(s^{1},s^{2},...,s^{t})\in R^{n\times t}$ to represent an input vector at a time t in MCS; the input series is multivariate and has several features at each time step. Hence, the proposed model pre-processes the input data as a three-dimensional tensor with multi-channels. The MSE of the entire time length is calculated using the loss function L. Meanwhile, the reconstruction loss is denoted by $L_{r}$ and the prediction loss $L_{p}$. The proposed autoencoder model aims to minimize the sum of both losses: $L_{s}=L_{r}+L_{p}.$ The pseudo-code for our adaptive temporal convolutional network autoencoder is shown in Algorithm 1.
**Algorithm 1:** Adaptive Temporal Convolutional Network Autoencoder (ATCN-AE)**Input:**X = model, dataset, labels**Output:**Malicious sensor data1.hidden_layers(h) = st = $\Theta =\left\{W,b\right\}$
2.reconstructed_output(y) = *h* = $\Theta^{\prime }=\left\{W^{\prime },b^{\prime }\right\}$
3.$W^{\prime }=WT=$ reconstructed_output(y)4.sensor_data_mse= mse.get_sensor_data($W^{\prime }=WT$)5.**for each** i in range (X,y) **do**6. Θ = sensor_data_mse[malicious_sensor_data_mse.len()−i]
7. predicted_labels = get_labels(mse,sensor_data)
8. st = get_st(labels,predicted_labels)
9. **if** malicious_sensor_data<st then10.  malicious_sensor_data = sensor_data
11. **endif**12.**end for**13.return malicious_sensor_data

#### 3.3.2. The Proposed ATCN-AE Architecture

**Figure 2:**
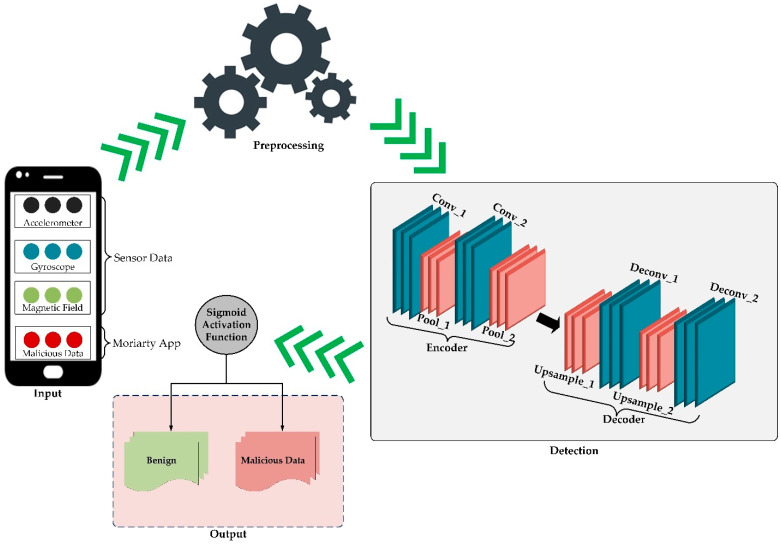
The architecture of the proposed adaptive temporal convolutional network autoencoder (ATCN-AE).

The proposed ATCN-AE comprises four components: the input, the preprocessing, the detection, and the output components, as displayed in Figure 2. The input component of the model architecture consists of data from the accelerometer, gyroscope, magnetic field sensors, and the Moriarty application, which is passed to the preprocessing component of the ATCN-AE architecture.

The proposed ATCN-AE model requires a labeled training dataset containing samples of both benign and malicious sensor data to learn effective detection capabilities. The malicious labels used during training can include known malicious activities like data integrity breaches or exploitation attempts. While the model is trained on recognized malicious activities, at test time, it can detect novel, unseen malicious operations that share similar underlying patterns with the malicious data seen during training. The embedding space learned by the autoencoder architecture allows for identifying anomalies that diverge from a benign population behavior. The target labels (0 for benign data and 1 for malicious data) were transformed into two-dimensional shapes of convolutional layers with two pooling layers for the encoder.

The detection component comprises the temporal convolutional layers, fully connected layers, pooling layers, convolutional layers, and upsampling layers. The temporal convolutional layers are part of the temporal convolutional network (TCN) component. They consist of dilated convolutional layers, which allow the network to capture temporal dependencies over a wide range of contexts. Dilated convolutions enable the model to learn relationships between sensor data points that are far apart in the sequence. The fully connected layers are used in both the encoder and decoder components of the autoencoder. These layers connect every neuron in one layer to every neuron in the next layer. In the encoder, fully connected layers are involved in compressing the input data into a latent space representation. The pooling layers are also part of the encoder component. They are used to downsample the feature maps generated by convolutional layers, reducing their spatial dimensions while retaining important features. In our architecture, they are employed before the fully connected layers to reduce dimensionality further.

On the other hand, the convolutional layers are used in both the encoder and decoder components. These layers apply convolution operations to input data, extracting features through filters or kernels. In the encoder, the convolutional layers contribute to feature extraction, while the decoder aids in reconstructing the original input data. The weights and biases of the convolutional layers of the proposed model were initialized. Then, the Adam optimization algorithm was set before the learning rate was defined as the hyperparameter. The input sequences were fed into the model, and the loss between the reconstructed output and the original input was computed. The Adam algorithm was used to backpropagate the gradients through the model before updating the model parameters. This process was repeated for 100 epochs to achieve convergence. Since the mean square error is calculated using benign data, it represents the error of the model. To this end, we set the maximum MSE as the threshold of the proposed autoencoder model.

To further enhance the performance of the ATCN-AE model, fine-tuning techniques were applied by adjusting the learning rate to optimize its performance on the specific task of malicious data detection in mobile crowd sensing. The aim was to improve the generalization capability of the ATCN-AE model. After training the model, we evaluated its performance on the test dataset by inputting the testing sequences into the trained model and computing the reconstructed output. We then calculated the loss between the reconstructed output and the original input sequences. The performance of the model was analyzed using evaluation metrics such as accuracy, precision, recall, and F1 score to see how well it detects malicious sensor data.

Meanwhile, the decoder component reconstructed the original input data from the compressed representation and progressively upsampled it to generate a reconstructed output. The decoder comprises a series of upsampling and two-dimensional deconvolutional layers, which receive outputs from the convolutional layers and pooling layers. The decoder output approximates the initial input fed into the encoder, forming a complete autoencoder system. Specifically, the upsampling layers are employed in the decoder to increase the spatial dimensions of the feature maps, effectively reconstructing the original input data from the compressed representation. These layers help restore the spatial information lost during the encoding process.

The proposed architecture incorporates adaptive learning mechanisms to adapt to the dynamic nature of the mobile crowdsensing environment. The adaptive learning feature helps improve the robustness of the model and ensures its effectiveness in detecting novel forms of malicious data in MCS. Furthermore, the TCN component captures temporal patterns and dependencies in the input data. It consists of two layers of temporal convolutional blocks. Each block typically includes a dilated convolutional layer, which allows the network to capture information from a wide range of temporal contexts. The dilated convolutions enable the model to learn dependencies between sensor data points far apart in the sequence. The TCN architecture is designed to extract relevant features from the input data, which the autoencoder component will further utilize. The output from the TCN is then passed through the encoder component, which captures the most important features of the input sequence in the sensor data while reducing its dimensionality.

The output component of the proposed ATCN-AE architecture consists of the sigmoid function. The sigmoid function breaks the input value between 0 and 1, mapping it to a probability-like output. It is employed in the formulated ATCN-AE model because of its effectiveness in binary classification where the goal is to assign an input instance to one of two classes (such as benign or malicious data). The output of the sigmoid function can be interpreted as the probability of the input belonging to the positive class (class 1—malicious data). A value closer to 1 indicates a higher probability of the input being classified as malicious data, while a value closer to 0 suggests a higher probability of the input being classified as benign data. To perform the binary classification, the ATCN-AE model employs a threshold value set at 0.5. If the output of the sigmoid function is greater than or equal to 0.5, the input is classified as belonging to the positive class (class 1—malicious data). Conversely, if the output is less than 0.5, the input is classified as belonging to the negative class (class 0—benign data).

By incorporating the sigmoid function in the output component of the ATCN-AE architecture, the model effectively maps the high-dimensional sensor data input to a probability-like output, enabling the binary classification of the data as either benign or malicious. This approach leverages the strengths of the sigmoid function in handling binary classification tasks, making it a suitable choice for the proposed model’s objective of detecting malicious data in mobile crowdsensing scenarios involving sensor data from mobile devices.

The model was developed and tested using Google Colab because of its flexible cloud-based environment. Implementation was performed in Python 3.11, leveraging the Keras deep learning library for its coding simplicity compared with TensorFlow. The training and evaluation were performed on an Intel Core i7 CPU (Intel, Santa Clara, CA, USA) with NVIDIA GeForce 940MX GPU (NVIDIA, Santa Clara, CA, USA) support, 16 GB RAM, and a Windows 10 64-bit operating system (Microsoft, Redmond, WA, USA). This computing configuration enabled efficient exploration and analysis of different model architectures and hyperparameters. The collaborative nature of the notebooks and the availability of GPUs allowed for rapid prototyping and experimentation. Overall, Google Colab provided an effective platform for implementing, iterating, and evaluating the proposed model.

## 4. Results and Discussion

In this section, we delve into the results obtained from our experiments and provide a comprehensive discussion of their implications. We analyze the performance of our proposed model, the adaptive temporal convolutional network autoencoder (ATCN-AE), in comparison with baseline methods. Through an in-depth examination of the findings, we aim to elucidate the strengths, weaknesses, and potential avenues for further improvement of our approach to detecting malicious data within mobile crowdsensing environments. We also present a comparative analysis of our experiment’s results against baseline approaches, serving as benchmarks to assess the efficacy of our model.

### 4.1. Performance Evaluation

The performance of the proposed ATCN-AE model is evaluated using a range of evaluation metrics, including accuracy, precision, recall, F1 score, and the area under the receiver operating characteristic curve (AUC-ROC). These metrics comprehensively assess how accurately the model classifies benign and malicious data instances. Table 4 presents the confusion matrix from the ATCN-AE.

**Figure 3:**
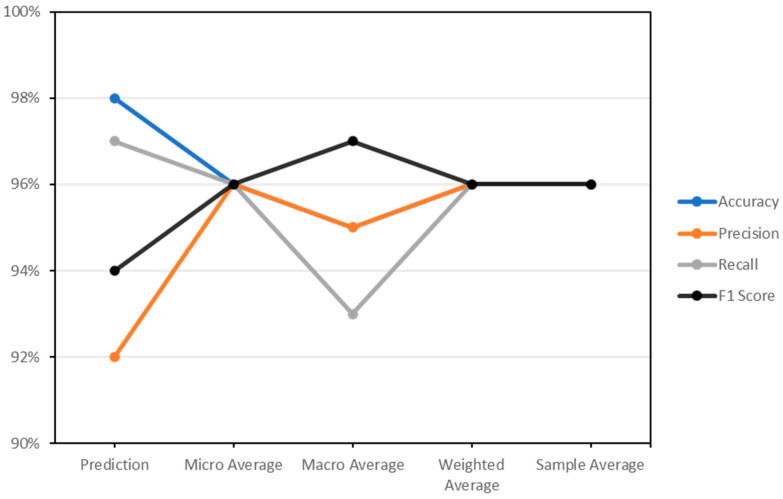
Evaluation metrics of the adaptive temporal convolutional network autoencoder (ATCN-AE).

From Table 4, the number of true positives represents the correctly classified malicious instances. In this case, the model correctly predicted 200 instances as malicious. False positives occur when the model predicts an instance as malicious when it is benign. In the confusion matrix, 16 instances are wrongly classified as malicious. False negatives arise when the model incorrectly classifies a malicious instance as benign. Here, six instances are falsely classified as benign. True negatives represent the instances that are correctly classified as benign. The model correctly predicted 1103 instances as benign. Furthermore, the evaluation of the proposed ATCN-AE using metrics including accuracy, precision, recall, F1 score, and the sample averages is shown in Table 5 and visualized in Figure 3.

The accuracy of the model is calculated by dividing the sum of true positives and true negatives by the total number of instances, as presented in Equation (3).

(3) $$\mathrm{Accuracy}=\frac{TN+TP}{TN+FP+TP+FN}$$

For this model, the accuracy is calculated as (1103+200)/(1103+6+200+16), resulting in an accuracy of 98%, which indicates that the model accurately classifies 98.4% of the instances in the dataset. This relatively high accuracy shows that the model distinguishes between benign and malicious data well. Precision measures the proportion of correctly identified malicious data out of all instances predicted as malicious, given by Equation (4).

(4) $$\mathrm{Precision}=\frac{TP}{TP+FP}$$

The precision is calculated as 200/(200+16), resulting in a precision of approximately 92%. This evaluation means that out of all instances predicted as malicious, around 92% are truly malicious. Recall, also known as sensitivity or true positive rate, measures the proportion of correctly identified malicious data out of all true malicious instances using Equation (5).

(5) $$\mathrm{Recall}=\frac{TP}{TP+FN}$$

The recall is calculated as 200/(200+6), resulting in approximately 97%, meaning the model captures 97% of the true malicious instances. F1 score, on the other hand, is the harmonic mean of precision and recall, providing a balanced measure of the performance of the model. It is calculated using Equation (6).

(6) $$F1\text{-}\mathrm{Score}=2\times \frac{Precision\times Recall}{Precision+Recall}$$

Using the calculated precision and recall values, the F1 score is approximately 94%. Micro, macro, weighted, and sample averages summarize the performance of the model across all classes. The micro average considers the total true positives, false positives, and false negatives of all the classes. The macro average calculates the average performance for each class without considering class imbalance. The weighted average considers the class distribution in the dataset, providing higher weightage to classes with more instances. The sample average is a simple average of the metrics for each class.

The micro average, accuracy, precision, recall, and F1 score are all 0.96, which indicates consistent performance across all classes. The macro average accuracy is 0.95, indicating good overall performance. However, the macro average recall is 0.93, slightly lower than the macro average precision of 0.95, indicating a potential imbalance in class performance. The weighted and sample averages are similar to the micro average, reflecting the balanced performance of the model.

Overall, the results indicate high accuracy and precision in detecting benign and malicious data. However, there is room for improvement in the precision and F1 score, suggesting that the model may benefit from further refinement to enhance its ability to capture all instances of malicious data. We further evaluate the prediction of the proposed ATCN-AE model based on the test loss, test mean absolute error, FPR, and TPR, as displayed in Table 6.

The test loss value of 0.171 indicates the average discrepancy between the model predictions and the true labels on the test dataset. The low test loss shows that predictions made by the model are closer to the ground truth labels, indicating better performance in capturing the patterns and features of the data. Meanwhile, the test MAE value of 0.96 represents the average absolute difference between the predictions on the test dataset and the true values. It measures the magnitude of the errors made by the ATCN-AE model in predicting malicious data. Within this evaluation, the low MAE indicates that the predictions made by the model are, on average, closer to the true values, demonstrating high accuracy in predicting malicious data.

Furthermore, the false positive rate is a performance metric that measures the proportion of incorrectly classified benign data out of all true benign data instances. For the ATCN-AE model, the FPR value of 0.013 indicates that only 1.3% of the true benign data are incorrectly classified as malicious by the ATCN-AE model. The low FPR is desirable as it signifies that the model effectively identifies benign data and minimizes false positives. On the other hand, the true positive rate quantifies the proportion of correctly identified malicious data out of all true malicious instances. Here, the TPR value of 0.873 indicates that the model correctly identifies 87.3% of malicious data. The higher TPR highlights that the model is effective, which, in the context of malicious data detection, can identify a significant portion of the true malicious data instances.

Another significant parameter of the ATCN-AE model is the reconstruction error. The threshold determines the sensitivity of the model to malicious data detection. Our experiment observed that the larger the threshold margin, the more malicious samples are misclassified as benign samples. Consequently, this decreases FPR and increases FNR. In contrast, a smaller threshold increases FPR and decreases FNR. Since we require the model to have a low false alarm, we set a significant threshold of 0.5 and obtained an FPR of 0.013.

**Figure 4:**
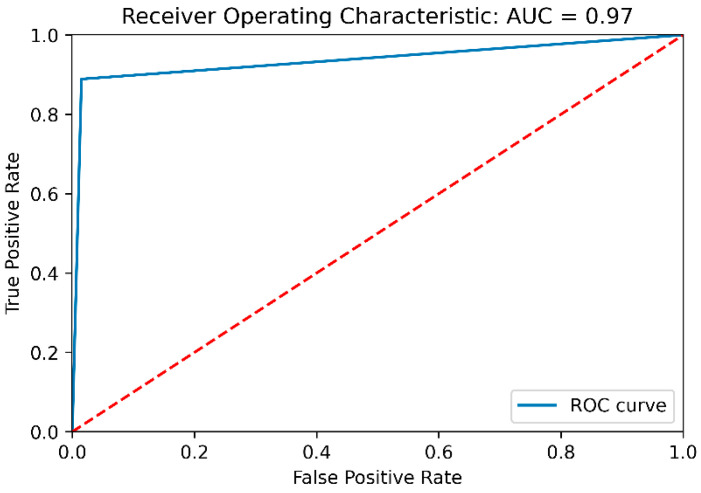
Receiver operator characteristics and the area under the curve.

The receiver operating characteristic (ROC) curve and the corresponding area under the curve (AUC) value of 0.97 provide a comprehensive assessment of the model’s ability to discriminate between benign and malicious data instances, as shown in Figure 4. The high AUC score demonstrates the model’s excellent discriminative power, further validating its effectiveness in the malicious data detection task.

**Figure 5:**
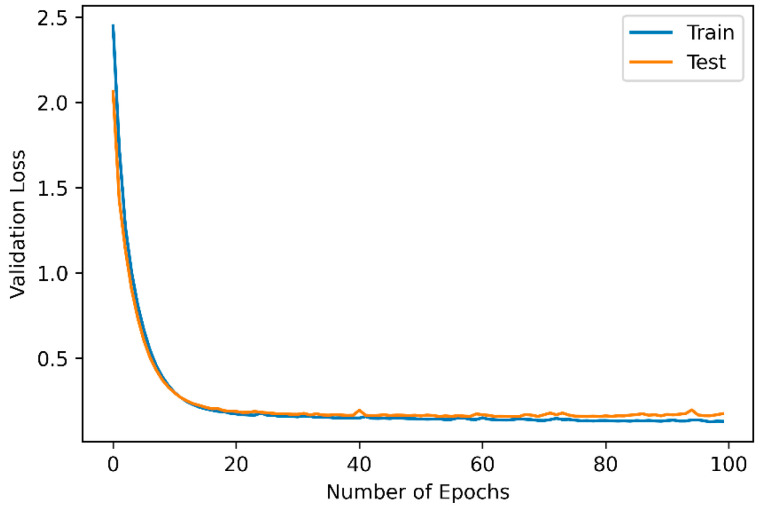
Validation loss during training and testing of the adaptive temporal convolutional network autoencoder (ATCN-AE).

Figure 5 depicts the loss and accuracy curves of the proposed model. As Figure 5 illustrates, the model learns and improves its performance; the loss decreases while the accuracy increases. This relationship between the loss and accuracy of the ATCN-AE model is a function of the training and validation process. We monitor the performance of the model using loss and accuracy on the validation dataset, which is calculated at the end of each training epoch. We trained the model with 100 epochs and obtained a decreasing loss and increasing accuracy before convergence during the training.

**Figure 6:**
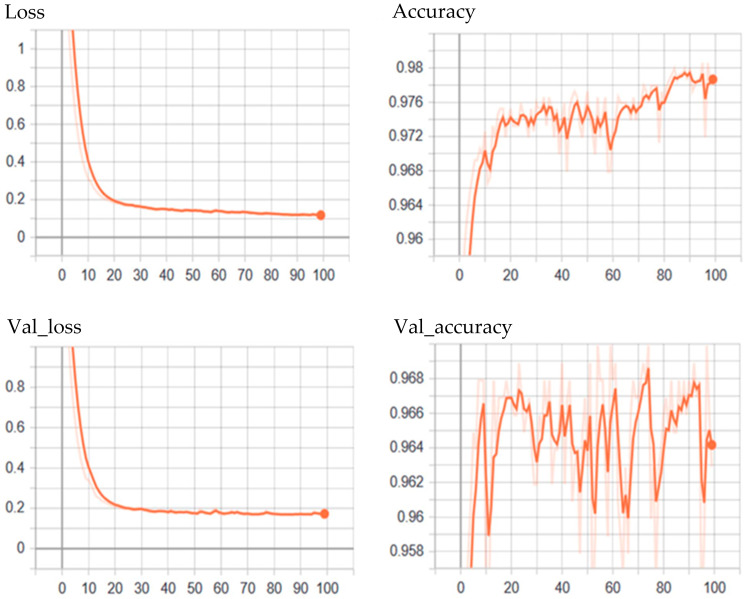
Evaluation of the adaptive temporal convolutional network autoencoder (ATCN-AE) on the training and testing dataset.

Comparing the validation loss and accuracy curves for both the training and testing datasets is crucial. As reflected in Figure 6, the validation loss decreases for both the training and testing datasets as the number of epochs increases. The validation loss on the training data shows consistent improvement while maintaining similar trends on the validation data, suggesting that the model is not overfitting and can generalize well to unseen sensor data. The low validation loss of 0.2 as the model converges at 100 epochs denotes a better performance, showing that the predictions by the model are closer to the true class. Meanwhile, the validation accuracy represents the percentage of correctly predicted instances in the validation dataset. It measures the classification performance of the model on unseen data. The high validation accuracy between 96.4% and 96.8% demonstrates that the model performs well and makes more accurate predictions even with fewer epochs.

**Figure 7:**
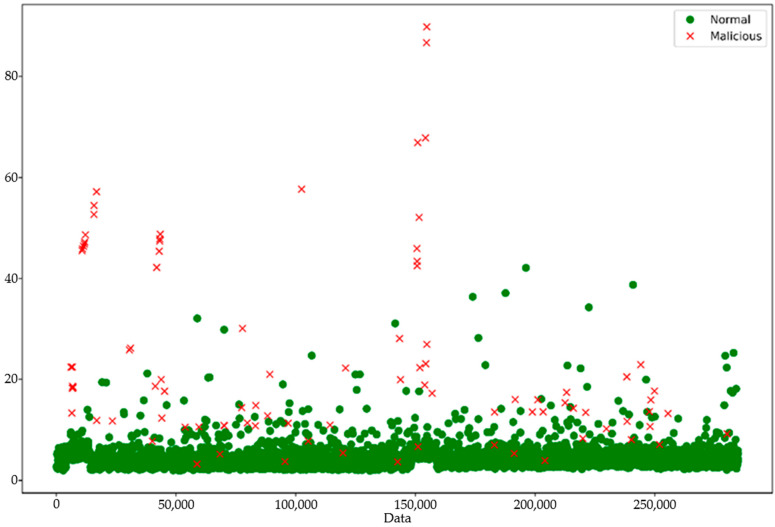
Malicious data detection by the proposed adaptive temporal convolutional network autoencoder (ATCN-AE) model.

The result in Figure 7 illustrates the predictions made by the proposed adaptive temporal convolutional network autoencoder (ATCN-AE) model on a dataset of mobile sensor data instances. The data points are classified as either “Normal” (benign) or “Malicious” based on the model’s output. The majority of the data points, represented by green markers, form a dense cluster near the bottom of the plot, indicating that the ATCN-AE model has classified these instances as benign sensor data conforming to expected patterns learned during training. Conversely, several data points marked with red “x” symbols are scattered at higher values along the y-axis, corresponding to instances identified as malicious by the model due to their significant deviation from the learned patterns of normal sensor data.

The clear separation between the two classes of data points in Figure 7 highlights the effectiveness of the ATCN-AE model in distinguishing between benign and malicious sensor data instances within the mobile crowdsensing environment. The data distribution reveals that the ATCN-AE model achieves a high level of accuracy in detecting malicious data instances while minimizing false positives and false negatives, as supported by the results in Table 6. The dense clustering of normal data points indicates accurate classification of benign sensor data, with only a few potential false negatives within the normal cluster. Similarly, the well-separated malicious data points show the effective identification of anomalous instances.

### 4.2. Comparison with State of the Art

**Figure 8:**
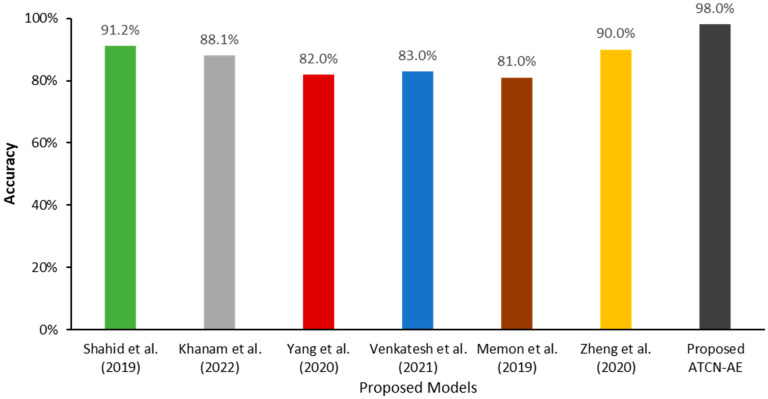
Comparison of the proposed ATCN-AE with existing detection models [15,16,19,28,29,30].

To benchmark the proposed adaptive temporal convolutional network autoencoder (ATCN-AE) against the existing literature, we compare its performance with several prior studies on the task of detecting malicious data within IoT and mobile crowdsensing environments. Specifically, our comparison focuses on similar autoencoder models and models that employ the SherLock dataset. Results presented in Table 7 demonstrate that our ATCN-AE model achieves approximately 98% accuracy in classifying benign and malicious data. This result signifies a marked improvement over existing baseline models such as the Sparse autoencoder (91.2%) [15], CFLVAE (88.1% accuracy) [16], and AE-D3F (82.0%) [19] as visualized in Figure 8. Additionally, our proposed model surpasses the performance of models utilizing the SherLock dataset, as evidenced by studies [28,29,30], achieving accuracies of 82%, 83%, and 81%, respectively.

The comparative evaluation highlights the capabilities of our ATCN-AE model in learning effective representations to detect anomalous patterns in crowdsensed data. Our proposed model outperforms previous approaches, underlining the significance of explicitly modeling temporal dependencies and leveraging deep learning for enhanced security in mobile crowdsensing systems.

Overall, the proposed adaptive temporal convolutional network autoencoder for malicious data detection model presents a promising solution to address the growing concern of detecting malicious data in mobile crowdsensing applications. By leveraging the temporal information, capturing relevant features with TCNs, compressing the data with autoencoders, and adapting to the dynamic nature of the environment, the proposed model aims to enhance the security and reliability of mobile crowdsensing systems.

The experimental results and evaluation confirm the effectiveness and potential of our ATCN-AE model in detecting malicious data in mobile crowdsensing environments.

The key novelty of the approach presented in this paper is the introduction of an adaptive temporal convolutional network autoencoder (ATCN-AE) model specifically designed for detecting malicious data in mobile crowdsensing (MCS) systems. The proposed model combines the strengths of temporal convolutional networks (TCNs) and autoencoders in a novel architecture to effectively capture and analyze temporal patterns in MCS data, enabling accurate identification of malicious activities.

Existing anomaly detection methods, such as those reviewed in the paper, have limitations when applied to MCS systems. Many approaches do not explicitly model the temporal dependencies present in the sensor data collected by mobile devices, which can be crucial for detecting malicious activities that may exhibit specific temporal patterns. Additionally, some methods are not adaptive and may struggle to keep up with the dynamic nature of MCS environments where data characteristics and attack vectors can evolve over time.

The proposed ATCN-AE incorporates an adaptive learning mechanism that enables the model to continuously update its parameters and adapt to new patterns or emerging malicious activities in the MCS environment. This adaptive nature enhances the model’s robustness and ensures its effectiveness over time, even as the characteristics of malicious data evolve. Unlike many existing anomaly detection models designed for general IoT or network traffic scenarios, the ATCN-AE is tailored specifically for the MCS domain. It is designed to handle the unique challenges of detecting malicious data in the context of mobile crowd sensing where sensor data from numerous devices needs to be analyzed for temporal patterns and anomalies.

The experimental results presented in the paper demonstrate the effectiveness of the ATCN-AE model in detecting malicious data in an MCS scenario, achieving an accuracy of 98% and outperforming existing baseline models. The comparative analysis highlights the significance of explicitly modeling temporal dependencies and leveraging deep learning for enhanced security in mobile crowdsensing systems.

## 5. Conclusions and Future Work

Mobile crowd sensing (MCS) has become an effective paradigm for large-scale sensing by engaging regular citizens. However, its open and distributed nature makes MCS systems highly susceptible to malicious data injection attacks that can severely compromise reliability. Developing capabilities to detect such malicious data accurately is therefore critical. This paper proposes a novel deep learning model called adaptive temporal convolutional network autoencoder (ATCN-AE) designed for enhanced malicious data detection in MCS-based systems. We introduce the hybridization of adaptive temporal convolutional networks (TCN) and autoencoders to effectively identify malicious data from mobile sensor data. The TCN component leverages dilated causal convolutions to capture long-range patterns and dependencies in the sensor data. It allows for the identification of the discriminative features indicating malicious activities. The autoencoder learns a compressed representation of the output from the TCN and reconstructs the original input. By comparing the reconstruction to the input, anomalies can be detected based on the error. Extensive experiments on the real-world SherLock dataset reveal the effectiveness of ATCN-AE, achieving 98% accuracy in classifying benign and malicious sensor data. Our comparative analysis highlights better performance over existing detection models.

In future work, we aim to expand the evaluation across more diverse MCS datasets. Additional temporal convolutional and autoencoder architectures can be explored to optimize detection capabilities further. Advancing MCS security through deep learning is an important research direction, and this paper makes promising strides. Robust crowdsensing systems will enable smarter cities, enhanced infrastructure monitoring, and novel context-aware services. While the proposed model focuses on detecting malicious data in mobile crowdsensing systems, many other types of sensors could be used in these systems, such as GPS, Wi-Fi, or Bluetooth. Future work could explore how to integrate these other types of sensors into the model. Also, the proposed model was evaluated using a large-scale dataset; it was not evaluated in a real-world setting. Future work could explore how to deploy the model in a real-world mobile crowdsensing system and evaluate its performance in that context. Last, the proposed model uses a combination of temporal convolutional blocks and an autoencoder; many other types of autoencoder architectures could be explored. This could include variational autoencoders (VAEs), generative adversarial networks (GANs), or other types of neural networks.

## Figures and Tables

**Table 1:** Summary of detection techniques used in existing anomaly data detection models.

Authors	Techniques	Dataset	Application Domain
Aloul et al. [14]	AAE	NSL-KDD	IoT
Shahid et al. [15]	Sparse AE	IoTPOT	IoT
Khanam et al. [16]	CFLVAE	NSL-KDD	IoT
Yang et al. [19]	AE	UNB 2017/MAWI	IoT
Lee et al. [20]	DAE	AWID	IoT
Mohammed et al. [24]	CDAE	AoI Readings	MCS
Alharam et al. [25]	DT/SVM/RF	Solar Radiation	MCS
Afzal-Houshmand et al. [26]	LSTM/SVM	SensingBus	MCS
Munoz-Organero et al. [27]	DBN/KNN/SVM	GPS Data	MCS
Venkatesh et al. [28]	Random Forest/DT/XGBoost	SherLock Dataset	MCS
Memon et al. [29]	SVM/LR/Naïve Bayes	SherLock Dataset	MCS
Zheng et al. [30]	Random Forest/ANN/LSTM	SherLock Dataset	MCS
Our Proposed Model	ATCN-AE	SherLock Dataset	MCS

**Table 2:** Selected files for use.

File Name	Sensors	Description
T2	Accelerometer Gyroscope Magnetic Field Orientation Rotation Vector Barometer	Samples collected for 4 s at 200 Hz Mean, median, and variance for each respective axis Covariance between axis, middle sample FFT components and their statistics An extracted subset features from orientation, rotation, and barometer sensors
Moriarty		The sampling occurs at the moment when the Moriarty malicious agent documents the clue. This includes the type of action and behavior, distinguishing between malicious and benign actions.

**Table 3:** Proposed adaptive temporal convolutional network autoencoder (ATCN-AE) notations.

Notations	Description
X	Input data
h	Hidden layer output
f	Mapping function
W	Weights matrix
b	Biases vector
Θ	Parameters {*W*, *b*}
σ	Activation function
y	Reconstructed output
$W^{\prime }$	Decoder weights matrix
$b^{\prime }$	Decoder biases vector
e	Reconstruction error
t	Input data sample
i	Output data sample
N	Number of samples
L	Overall loss function
Lr	Reconstruction loss
Lp	Prediction loss
st	Input vector at time t
MSE	Mean squared error

**Table 4:** Confusion matrix from the adaptive temporal convolutional network autoencoder (ATCN-AE).

		Predicted Class	
		Benign (0)	Malicious (1)
True class	Benign (0)	1103 (*TN*)	6 (*FP*)
	Malicious (1)	16 (*FN*)	200 (*TP*)

**Table 5:** Evaluation metrics of the adaptive temporal convolutional network autoencoder (ATCN-AE).

	Accuracy	Precision	Recall	F1 Score
Prediction	0.98	0.92	0.97	0.94
Micro Average	0.96	0.96	0.96	0.96
Macro Average	0.95	0.95	0.93	0.97
Weighted Average	0.96	0.96	0.96	0.96
Sample Average	0.96	0.96	0.96	0.96

**Table 6:** Evaluation metrics of the adaptive temporal convolutional network autoencoder (ATCN-AE).

Prediction	Score
Test loss	0.171
Test MAE	0.960
FPR	0.013
TPR	0.873

**Table 7:** Comparison of the proposed ATCN-AE with existing detection models.

Detection Models	Accuracy
Shahid et al. [15]	91.2%
Khanam et al. [16]	88.1%
Yang et al. [19]	82.0.%
Venkatesh et al. [28]	82.0%
Memon et al. [29]	83.0%
Zheng et al. [30]	81.0%
Proposed ATCN-AE	98.0%

## Data Availability

The authors of the SherLock and Moriarty dataset [31] will grant access to the dataset upon request.
